# Bird Dispersal as a Pre-Adaptation for Domestication in Legumes: Insights for Neo-Domestication

**DOI:** 10.3389/fpls.2019.01293

**Published:** 2019-10-15

**Authors:** Hester Brǿnnvik, Eric J. von Wettberg

**Affiliations:** ^1^Department of Plant and Soil Science, University of Vermont, Burlington, VT, United States; ^2^Mathematical Biology and Bioinformatics Laboratory, Peter the Great St. Petersburg Polytechic University, Saint Petersburg, Russia

**Keywords:** neodomestication, bird dispersal, legumes (fabaceae), preadaptation, natural history, domestication syndrome, seed dispersal

The first chapter of Darwin’s *On the Origin of Species* famously used examples of human selection of domesticated plants and animals to lay the groundwork for the Theory of Evolution (Darwin, 1859). Since at least the works for Darwin (1868), domestication of plants and animals have been used as major examples of strong selection radically altering the morphology, architecture, and behavior of organisms on which our contemporary society relies for food, fiber, and fuel. Consequently, it is not surprising that crop domestication remains a vibrant area of research. Despite this ongoing interest in the field of domestication, we greatly lack ecological and natural history studies of crop wild relatives in their wild settings.

One instance of the great lack of natural history of crop wild relatives is in understanding, the dispersal biology of crop wild relatives as a potential pre-adaptation for domestication. The domestication of annual crops such as cereals and grains legumes is thought to require two key traits that are part of the domestication syndrome (Hammer, 1984): seeds that lack dormancy and fruit structures that are indehiscent (Purugganan and Fuller, 2009; Ogutcen et al., 2018; Smýkal et al., 2018). Debate in the domestication literature has long been generally assumed that both of these traits are disfavored in natural populations and then speculated whether the genetic changes underlying the domestication shift arose from standing variation at low frequency in natural populations (mildly deleterious alleles) or new mutations (e.g., Morrell et al., 2012; Olsen and Wendel, 2013; Gaut et al., 2018; Hufford et al., 2019; Lye and Purugganan, 2019). However, the assumption that these traits would be disfavored in natural populations is just that and is not based on careful observation of actual dispersal or germination biology. We think it is possible that these traits may, in some, taxa have evolved long ago as part of their dispersal biology, and that this possibility has not been sufficiently studied. Birds and other animal dispersers may more effectively disperse whole fruit than individual seeds, favoring indehiscence in legumes—but we do not know this for a critical lack of natural history knowledge. If so, this is a pre-adaptation for domestication which may have pre-disposed some groups toward domestication and can be used in selected taxa for neodomesticaiton. Here, we document the limited natural history knowledge of bird dispersal in legumes that are consumed by humans and show a significant gap in our natural history knowledge of crop wild relatives.

Animal dispersal syndromes in plants are well described—for example, by the landmark review by Howe and Smallwood (1982). Birds can be particularly effective at dispersing seeds a great distance and to suitable habitats. Bird dispersal can be critical to allow offspring to escape higher disease pressure by escaping the proximity of parents. Many bird dispersal behaviors also allow directed dispersal to particular habitats where seedling establishment is more likely to be successful, whether by burying seeds through caching behavior or dropping consumed seeds with fertilizing feces. However, birds are not the only effective animal dispersers of seeds. For herbaceous plants or grasses that occur in habitats where bird dispersal may not be effective, large mammalian herbivory may be a more common form of long-distance dispersal (Janzen, 1984). Ants are also often effective dispersers, particularly in temperate environments, although they can also be significant seed predators (e.g., Hulme, 1998).

To see if animal dispersal vectors for a suite of crops are well done, we performed a literature search using the terms “bird” and “dispersal” and a list of cultivated legumes from Smýkal et al. (2014) and our own knowledge, to find published or gray-literature reports of bird dispersal of legumes in genera known to have cultivated species. In our literature search, we were only able to uncover four instances of descriptions of bird dispersal in genera with cultivated legumes (**Table 1**). This is a rather small number of reports, particularly given the very large size of both of the *Fabaceae* and the high number of cultivated species in the family. This search approach almost certainly misses reports in languages other than English but is sufficient to make our primary point very clearly, which there is an absence of important natural history work characterizing crop wild relatives.

**Table 1:** Examples of bird dispersal in legumes.

Location	Plant	Disperser	Evidence	Reference
Veracruz, Tamaulipas, and Oaxaca, Mexico	*Acacia cornigera*	*Psilorhinus morio*	Germination from fecal samples	Janzen (1969)
Muak-lek, Thailand	*Acacia mangium* Willd. × *A. auriculiformis* A. Cunn. ex Benth.	“Small birds”	Observed consumption	Sornsathapornkul and Owens (1998)
Bolivian Andes	*Inga feuillei*	*Thectocercus acuticaudatus*	Observed stomatochory	Blanco et al. (2015)
Western Australia	*Medicago* *sp*.	*Dromaius novaehollandiae*	Found in fecal sample	Calviño-Cancela et al. (2006)
Sumba, Indonesia	*Phaseolus lunatus*	*Cacatua sulphurea citrinocristata*	Observed consumption	Hidayat (2014)
Junín, Peru	*Phaseolus vulgaris*	“Pichupa”		Tohme et al. (1995)
“Home range”	*Pueraria lobata*	“Birds”		EPPO (2007)

These examples do show some important observations. In *Phaseolus*, bird dispersal is likely widespread, and likely a key component of the very large distribution of wild *Phaseolus vulgaris* (and potentially *Phaseolus lunatus,* lima bean) from Mexico to the central Andes of South America (Ariani et al., 2017). This broad distribution likely contributed to the two independent domestications of common bean (and lima bean). Although the possibility bird dispersal has been remarked upon (Gepts, pers. comm.), there are not many natural history observations of wild *Phaseolus* to determine how widespread bird dispersal is, whether different guilds of birds are responsible for it, or whether *Phaseolus* species vary in their propensity for bird dispersal.

Understanding whether birds are a disperser is a starting point for understanding dispersal syndromes in the wild. As a pre-adaptation for cultivation and domestication, the pod would need to be indehiscent. This is a trait that can be tested in a common garden, although dehiscence can be modulated by the environment, such that the humidity of the environment may affect dehiscence (e.g., Lush et al., 1980; Ogutcen et al., 2018). Consequently, dehiscence in a humid environment may not indicate dehiscence in an arid environment.

Most taxa, particularly those in parts of Africa, South, Southeast, Southwest, and East Asia, and South America, where natural history observations published in English may be particularly absent, likely are simply data deficient. Given that the majority of legumes were domesticated in Vavilovian centers of origin in these regions, certainly there is almost simply a great lack of natural history data.

There is a great need for more ecological study and natural history observation of crop wild relatives. Crop wild relatives are the most significant reservoir of adaptative variation for providing disease resistance, abiotic stress tolerance, and other important traits to cultivated species. As crop wild relatives receive almost no conservation protection in natural populations and are badly underrepresented in most Genebanks (Maxted and Kell, 2009; Warschefsky et al., 2014), natural history study of these species remains critical to their long-term conservation.

## Author Contributions

Both authors contributed to the idea development, literature search, and writing of this mini-review.

## Funding

The literature review portion of this work was supported by Russian Scientific Fund Project No. 18-46-08001 on the basis of a unique scientific installation «Сollection of plant genetic resources VIR», by a cooperative agreement from the United States Agency for International Development under the Feed the Future Program AID-OAA-A-14-00008 to D.R.Cook and Co-PI EW, by a grant from the US National Science Foundation Plant Genome Program under Award IOS-1339346 to D.R.Cook, and EW; US NIFA grant # 2018-67013-27619 R. Varma Penmetsa and EW. EW is further supported by the USDA Hatch program through the Vermont State Agricultural Experimental Station.

## Conflict of Interest

The authors declare that the research was conducted in the absence of any commercial or financial relationships that could be construed as a potential conflict of interest.
